# FLOURISHCARE: HELPING OLDER ADULTS FLOURISH IN RURAL COMMUNITIES

**DOI:** 10.1093/geroni/igz038.1024

**Published:** 2019-11-08

**Authors:** Anna Faul, Joe D’Ambrosio, Pamela Yankeelov, Samantha G Cotton, Megan Austin, Mona Huff, Darla Handy

**Affiliations:** University of Louisville, Louisville, Kentucky, United States

## Abstract

Flourishing represents living within an optimal range of human functioning. To foster older adults flourishing, our FlourishCare model of care coordination was developed as part of a HRSA Geriatric Workforce Enhancement Program (GWEP) to transform PC sites by delivering coordinated services in 6 rural KY counties. FlourishCare is unique in its integration of academic teams, community health teams and mental health specialists within age-friendly primary care health systems.FlourishCare recognizes that many of the largest drivers of health care costs fall outside the clinical care environment. One of the major components of Flourish is the involvement of community health navigators (CHNs), community education coordinators (CECs) and community coalitions working with the health teams to respond to the social determinants of health. For each patient that is referred and agrees to participation in the program, the CHN performs Flourish clinical and home assessments and obtains medical records. A repeated measure designed was used to assess the 60 patients who have baseline assessments results along with at least a 6-month assessment and in some cases a 12-month assessment (n= 20). Patients have demonstrated a significant increase in their flourishing across all determinants of health. There was also a significant improvement the total FI from pre to post-test at 6-month. These outcomes illustrate that it is important to operationalize the outcomes for older adults by evaluating the success as the ability of the older adult to flourish when they can maintain current functioning, access to resources have sufficient support systems in place.

